# Age-Related Hearing Loss With Tinnitus and Physical Frailty Influence the Overall and Domain-Specific Quality of Life of Chinese Community-Dwelling Older Adults

**DOI:** 10.3389/fmed.2021.762556

**Published:** 2021-10-21

**Authors:** Weibin Zhang, Jian Ruan, Ruxin Zhang, Min Zhang, Xiuhua Hu, Zhuowei Yu, Zhao Han, Qingwei Ruan

**Affiliations:** ^1^Laboratory of Aging, Anti-aging & Cognitive Performance, Shanghai Institute of Geriatrics and Gerontology, Fudan University, Shanghai, China; ^2^Department of Geriatrics, Shanghai Key Laboratory of Clinical Geriatrics, Huadong Hospital, and Research Center of Aging and Medicine, Shanghai Medical College, Shanghai Institute of Geriatrics and Gerontology, Fudan University, Shanghai, China; ^3^Department of Otolaryngology, Huadong Hospital, Shanghai Medical College, Fudan University, Shanghai, China; ^4^Department of Geriatrics, Huadong Hospital, Shanghai Medical College, Fudan University, Shanghai, China

**Keywords:** age-related hearing loss, tinnitus, physical frailty, health-related quality of life, age-related hearing loss with tinnitus

## Abstract

**Objective:** To investigate the impact of the severity of age-related hearing loss (ARHL) and tinnitus, presence of ARHL and/or tinnitus, and physical frailty on the health-related quality of life (HRQoL) and domain-specific HRQoL in Chinese community-dwelling older adults.

**Design:** This was a cross-sectional study of a community-based cohort.

**Participants:** We evaluated Chinese older adults (*n* = 429, 183 men and 246 women) aged ≥ 58years.

**Measurements:** The severity of HL and tinnitus were measured using pure-tone audiometry and the Tinnitus Handicap Inventory (THI), respectively. Physical frailty was measured using the five-item Fried scale. HRQoL was assessed using the Assessment of Quality of Life-8-Dimension (AQoL-8D) multi-attribute utility instrument (35 HRQoL items and eight domain-specific HRQoL subcategories). Low HRQoL (HRQoL score or subscores in the highest quintile) was used as a dependent variable in logistic regression analyses adjusted for demographic (Model 1) and health-related (Model 2) and psychosocial (Model 3) confounders.

**Results:** Age-related hearing loss severity was an independent determinant of senses in the physical dimension of HRQoL after adjusting for all covariates. Tinnitus severity was significantly associated with HRQoL and with independent living, senses, and pain in the physical dimension after adjusting for demographic and health-related covariates and was still associated with independent living and senses after adjusting for all covariates. The presence of ARHL and/or tinnitus was significantly associated with independent living and senses in the physical dimension after adjusting for all the covariates. Physical frailty was an independent determinant of HRQoL, independent living, and pain in the physical dimension and with mental health, happiness, and coping in the psychosocial dimension after adjusting for demographic and health-related covariates. The association with HRQoL, independent living, and pain in the physical dimension, and with happiness and coping in the psychosocial dimension remained significant after adjusting for the covariates. Depressive symptoms, social dysfunction, and a number of comorbidities were critical determinants of psychosocial HRQoL.

**Conclusion:** Physical frailty has a stronger and more profound effect on HRQoL, particularly on independent living and pain in the physical dimension and on happiness and coping in the psychosocial dimension. Domain-specific HRQoL should be considered in the management of patients with ARHL with tinnitus and physical frailty.

**Clinical Trial Registration:** www.ClinicalTrials.gov, identifier: NCT2017K020.

## Introduction

Age-related hearing loss (ARHL) is the most common chronic disorder of the sensory system (1). Physical frailty, especially mobility frailty characterized by slowness and/or weakness, is a common geriatric syndrome associated with a decline in the functioning of multiple physiological systems (2, 3). Both ARHL and physical frailty are principal causes of chronic disability (2, 4) and are expected to increase with the rapid growth of the aging population. Hearing loss is projected to become one of the top 10 burdens of disease by 2030 (5). ARHL is associated with frailty, falls, poor psychosocial well-being (e.g., social isolation, loneliness, and depression), poor communication, and cognitive decline (3, 6–8). Chronic subjective tinnitus is another common disorder of the auditory system that occurs as a comorbidity of ARHL in older adults.

The severity of ARHL (9) and tinnitus (10), and the presence of ARHL and tinnitus (11) have been reported to be associated with poor health-related quality of life (HRQoL). Physical frailty is an emerging global health burden, affecting ~11% of older community-dwelling adults in high-income countries and having an even higher prevalence in low-income countries (2, 12). Physical frailty could result in various adverse outcomes, such as disability, falls, cognitive decline, depression, lower HRQoL, hospitalization, and mortality (2, 13, 14). Although ARHL, tinnitus, and physical frailty are important determinants of HRQoL, differences in their effects on HRQoL and on specific physical and psychosocial dimensions of HRQoL remain poorly understood.

The negative effect of the severity of ARHL and tinnitus and the presence of ARHL and/or tinnitus on HRQoL varies according to the assessment instruments used (e.g., hearing-related QoL and generic QoL instruments) and confounders (15, 16). Overall, HRQoL with broader dimensions is more widely used than hearing-related QoL to assess the physical and mental health of patients with ARHL and tinnitus. A series of cross-sectional studies on a Korean adult population assessed HRQoL using the EuroQol five-dimensional (EQ-5D) questionnaire, and those with moderate to severe hearing loss had lower health status than those without hearing loss after adjusting for sociodemographic, health-related, and psychological factors, and comorbidities (9). Moreover, patients with hearing loss with tinnitus have lower mobility, usual activity, and anxiety/depression HRQoL than those with a normal hearing without tinnitus (17). In studies that assessed the overall HRQoL of patients with hearing loss and tinnitus using the Medical Outcomes Study 36-item Short-Form Health Survey (12-item) (10, 18), individuals with more severe tinnitus had worse physical, bodily pain, vitality, and mental health scores (18). Further, sex, depressive symptoms, anxiety, and somatization symptoms were found to regulate the effect of chronic tinnitus on the mental and physical dimensions of HRQoL (10).

The older population has more complex multimorbidity due to the overlap among physical frailty, comorbidity, and chronic physical and mental health diseases (19). Physical frailty is highly prevalent and usually coexists with ARHL and tinnitus in older adults. Importantly, physical frailty might be a potential mediator of the association between ARHL with tinnitus and HRQoL (6). The effect of ARHL on cognition is greater among individuals older than 75 years (20). Furthermore, the coexistence of physical frailty with ARHL has been confirmed to be associated with worse cognitive performance in the elderly (21). Many previous studies have shown that community-dwelling older people with physical frailty have worse HRQoL and specific HRQoL domains after adjusting for confounders (13, 14). Thus, the impact of the severity of ARHL and tinnitus, and the presence of ARHL and/or tinnitus and physical frailty on the low HRQoL and specific domains of HRQoL needs to be further investigated.

This study aimed to determine whether these associations were different and independent of demographic, health-related, and psychosocial factors. We hypothesized that the association of physical frailty with HRQoL and specific domains in community-dwelling older people is different from that of ARHL with tinnitus.

## Materials and Methods

### Study Design

In total, 429 community-dwelling Chinese older adults (age ≥58 years) were recruited from our previous cross-sectional cohort study on ARHL with tinnitus and general health (22). The number and characteristics of participants were the same as those in our recently published article describing the association of frailty phenotypes with age-related hearing loss and tinnitus (3).

### Participants

All the participants underwent cognitive function, hearing and tinnitus severity, QoL, and comprehensive geriatric assessments. The inclusion criteria were: (1) permanent residence in the community, (2) age ≥58 years, and (3) willingness to participate in the project. The exclusion criteria were as follows: (1) handicap, (2) profound loss of hearing (including hearing aids) and vision, (3) dementia, (4) psychiatric/neurological disorders and dyslexia, and (5) taking medicines that affect cognitive performance. All participants provided informed consent prior to participation. This study was approved by the Ethics Committee of Huadong Hospital (Approval No. Ref 2018K055), and was conducted in accordance with the Declaration of Helsinki.

### HRQoL Assessment

Health-related quality of life was assessed using the 35-item Assessment of Quality of Life (AQoL-8D) self-report questionnaire. The questionnaire covers three physical (independent living, pain, and senses) and five psychosocial dimensions (mental health, happiness, coping, relationships, and self-worth) (23). The senses domain was defined as a sensory system function. The respondents were asked to rate each item, and the HRQoL score was calculated by summing answers to the 35 QoL items (from 0 to 140). The unweighted total score and subscore of each specific domain were standardized, and the total HRQoL score ranged from 0 to 100, with lower scores indicating higher HRQoL. The highest quintile for the HRQoL score, as well as each domain-specific HRQoL subscore, was defined as 1 = low HRQoL, while scores below the highest quintile were defined as 0 = better HRQoL.

### Assessment of ARHL, Tinnitus, Cognition, and Physical Frailty

The severity of ARHL and chronic subjective tinnitus (i.e., >3 months) was assessed using pure-tone audiometry and the Tinnitus Handicap Inventory (THI), respectively. The pure-tone average (PTA) in the better ear was calculated using the.5-, 1-, 2-, and 4-kHz thresholds. The participants were then divided into four groups based on their hearing levels (0 = normal hearing [PTA ≤ 15 dB]; 1 = subclinical hearing loss [PTA >15, ≤ 25 dB]; 2 = mild loss [PTA > 25, ≤ 40 dB]; and 3 = moderate to severe hearing loss [PTA > 40 dB]) (24).

Tinnitus was classified into three categories by severity [0 = without tinnitus, 1 = mild (THI score: 1–16 and 18–36); 2 = moderate (THI score: 38–56, 58–76), and 3 = severe (THI score: 78–100)]. The patients were also scored based on the presence of ARHL and/or tinnitus (0 = without ARHL and tinnitus; 1 = only ARHL; 2 = only tinnitus; and 3 = with ARHL and tinnitus).

Cognitive performance was assessed using the global cognitive screening test; Mini-Mental Status Exam (MMSE); and normative z-scores of the neuropsychological test battery, including executive or attention, language, and memory domains (3, 25). Physical frailty was evaluated using the five-item Fried scale (weakness, slowness, unexplained weight loss, fatigue, and low physical activity) with Chinese reference values (26, 27). The Fried scale score ranges from 0 to 5, with scores 3–5 and 1–2 indicating frail and prefrail, respectively.

### Covariates

All the subjects underwent face-to-face interviews to collect demographic information and completed a standardized medical questionnaire. The demographic information included age, sex, education level, height, body weight, and body mass index (BMI, calculated as weight in kg divided by height in m^2^). The medical questionnaire included self-reported smoking, alcohol intake, and chronic comorbidities (3). The number of comorbidities was categorized as 0, 1, 2, and >2. Chronic diseases, such as diabetes mellitus, cardiovascular disease (CVD), stroke, and non-skin malignancies were also considered as covariates. Depressive symptoms, neuropsychiatric symptoms, and social dysfunction were assessed using the 15-item short form of the Geriatric Depression Scale, a brief version of the self-report Neuropsychiatric Inventory Questionnaire (NPI), and the 21-item Social Dysfunction Rating Scale, respectively (3).

### Statistical Analysis

Continuous variables were expressed as medians and quartiles, and non-normally distributed variables were tested using the Kolmogorov–Smirnov test. Meanwhile, categorical data were expressed as proportions and compared using the *X*^2^ test. Differences in continuous and categorical variables between groups were analyzed using Spearman's test for bivariate correlation and one-way analysis of variance, respectively. A univariate correlation was analyzed using the test of homogeneity or the Mann–Whitney *U* test as appropriate. The association of the severity of ARHL, tinnitus, or the presence of ARHL with tinnitus and HRQoL and specific domains of HRQoL was assessed using multiple logistic regression analyses adjusted for age, sex, education level (Model 1); adjusted for covariates in Model 1 and other health covariates (Model 2); and adjusted for covariates in Model 2 and psychosocial factors, including social dysfunction, depression, and neuropsychiatric symptoms (Model 3). These models were run for low HRQoL scores and then separately for each low domain-specific subscore, which was used as a dependent variable. All the statistical analyses were performed using SPSS version 18.0, and *P* < 0.05 was considered statistically significant.

## Results

### Influencing Factors of HRQoL and/or Tinnitus

Patient characteristics stratified by ARHL, tinnitus, and the presence of ARHL and tinnitus are shown in Table 1. Bivariate analysis showed that sex, tinnitus severity, physical frailty, comorbidity number, CVD, diabetes mellitus, social dysfunction, depressive symptoms, and NPI were significantly associated with HRQoL. The severity of ARHL and tinnitus, the presence of ARHL and/or tinnitus, physical frailty, comorbidity number, CVD, stroke, social dysfunction, age, depressive symptoms, and NPI were significantly associated with the physical dimensions of HRQoL. Meanwhile, tinnitus severity, physical frailty, comorbidity number, CVD, diabetes mellitus, smoking, age, social dysfunction, depressive symptoms, and NPI were significantly associated with the psychosocial dimensions of HRQoL.

**Table 1:** Participant characteristics by severity of ARHL and tinnitus and the presence of ARHL and/or tinnitus.

		**ARHL severity** ***N*** **=** **425**						**Tinnitus severity N** **=** **427**					**The presence of ARHL and/or tinnitus N** **=** **424**					
	**Total sample**	**0**	**1**	**2**	**3**	**F/X^**2**^value**	**p**	**0**	**1**	**2**	**F/X^**2**^value**	**P**	**0**	**1**	**2**	**3**	**F/X^**2**^value**	**p**
	***N* = 429**	***N* = 105**	***N* = 78**	***N* = 125**	***N* = 117**			***N* = 279**	**N = 108**	**N = 40**			**N = 77**	**N = 140**	**N = 31**	**N = 176**		
**Age**	72.91 ± 7.014	71.30 ± 6.262	69.92 ± 6.279	73.50 ± 7.490	75.52 ± 6.488	13.456	<0.01	72.90 ± 6.672	72.94 ± 7.350	72.47 ± 8.290	0.071	0.932	72.12 ± 6.287	73.21 ± 6.683	69.32 ± 5.918	73.49 ± 7.476	3.609	0.013
**Female**	246 (57.30%)	63 (60.00%)	49 (62.82%)	74 (59.20%)	58 (49.57%)	4.325	0.228	161 (57.71%)	64 (59.26%)	20 (50.00%)	1.059	0.589	48 (62.34%)	78 (55.71%)	17 (54.84%)	101 (57.39%)	1.011	0.799
**Education**	12 (9, 15)	12.0 (9.0, 16.0)	11.5 (9.0, 15.0)	12.0 (9.0, 14.0)	12.0 (9.0, 15.0)	1.944	0.122	12.0 (9.0, 15.0)	11.0 (9.0, 14.0)	12.0 (9.0, 15.0)	0.752	0.472	12.0 (9.0, 16.0)	11.5 (9.0, 15.0)	12.0 (9.0, 16.0)	12.0 (9.0, 15.0)	2.298	0.077
**BMI**	23.2 (16.4, 25.7)	24.7 (21.7, 26.4)	23.300 (20.575, 25.625)	22.5 (13.0, 25.2)	19.95 (11.75, 25.30)	14.304	<0.01	23.30 (19.40, 25.70)	22.00 (12.00, 25.40)	21.35 (15.25, 26.25)	2.897	0.056	24.10 (21.45, 26.10)	22.60 (16.00, 25.25)	25.00 (23.30, 27.70)	22.00 (12.00, 25.30)	7.799	<0.01
**Smoking** (%)						5.136	0.526				2.396	0.662					5.407	0.482
Never smokers (0)	353 (82.3%)	90	65	106	89			235	85	32			68	114	24	144		
Ever smokers (1)	38 (8.9%)	9	5	10	13			21	11	5			6	13	4	13		
Current smokers (2)	31 (7.2%)	5	8	7	11			19	9	3			2	10	3	16		
**Drinking** (%)						2.132	0.922				3.752	0.411					9.405	0.119
Never drinkers (0)	378 (88.1%)	95	67	111	101			251	91	34			70	126	27	150		
Ever drinking (1)	14 (3.3%)	3	4	4	3			7	5	2			3	1	0	10		
Current drinking (2)	30 (7.0%)	6	7	8	9			17	9	4			3	11	4	12		
**Comorbidity**						5.380	0.800				4.024	0.676					2.523	0.112
0	49 (11.4%)	13	11	12	13			35	12	2			12	17	1	19		
1	127 (29.6%)	38	21	33	34			85	28	13			26	41	12	47		
2	124 (28.9%)	26	22	43	31			81	32	10			18	46	11	46		
>2	98 (22.8%)	24	15	31	27			59	27	12			18	26	6	47		
**CVD**						8.761	0.033				1.155	0.561					0.709	0.871
0	174 (40.6%)	45	37	37	53			109	45	19			34	54	12	72		
1	254 (59.2%)	60	41	87	64			170	62	21			43	86	19	103		
**Diabetes mellitus**						3.160	0.368				0.773	0.679					2.170	0.534
0	352 (82.1%)	87	69	99	93			232	88	31			62	119	27	139		
1	76 (17.7%)	18	9	25	24			47	19	9			15	21	4	36		
**Stroke**						1.305	0.728				1.453	0.510					2.295	0.507
0	386 (90%)	94	73	112	104			248	98	38			67	126	30	159		
1	42 (9.8%)	11	5	12	13			31	9	2			10	14	1	16		
**Non-skin malignancy**						1.244	0.743				2.401	0.301					0.628	0.920
0	397 (92.5%)	99	73	115	106			262	98	35			73	129	29	161		
1	31 (7.2%)	6	5	9	11			17	9	5			4	11	2	14		
	**Total sample**	**0**	**1**	**2**	**3**	**F/X** ^**2**^ **value**	**p**	**0**	**1**	**2**	**F/X** ^**2**^ **value**	**P**	**0**	**1**	**2**	**3**	**F/X** ^**2**^ **value**	**p**
	***N*** **=** **429**	***N*** **=** **105**	***N*** **=** **78**	***N*** **=** **125**	***N*** **=** **117**			***N*** **=** **279**	**N** **=** **108**	**N** **=** **40**			**N** **=** **77**	**N** **=** **140**	**N** **=** **31**	**N** **=** **176**		
**Physical frailty stratification**						16.250	0.012				3.655	0.455					3.703	0.719
Robust (0)	176 (41%)	48	41	52	34			119	44	13			32	57	16	70		
Pre-physical frailty (1)	202 (47.1%)	50	32	54	64			126	54	20			40	62	13	84		
Physical frailty (2)	45 (10.5%)	7	4	17	17			29	9	7			5	17	2	21		
**MMSE**	28 (26, 29)	28.0 (27.0,29.0)	28.0 (26. 0,29.0)	27.0 (26.0, 29.0)	27.0 (26.0, 28.0)	4.761	0.003	28.0 (26.0,29.0)	27.0 (26. 0,28.0)	27.0 (26.0, 28.0)	0.979	0.377	28.0 (26.0, 29.0)	27.0 (26.0,29.0)	28.0 (27.0, 29.0)	27.0 (26.0, 29.0)	2.389	0.06
**Cognitive stratification**						47.692	<0.01				5.497	0.240					23.836	<0.001
Normal Pre-MCI MCI	215 (50.1%) 111 (25.9%) 103 (24%)	72 22 11	46 25 7	54 36 35	42 27 48			150 70 59	47 30 31	17 10 13			52 17 8	66 35 39	22 6 3	73 52 51		
**Social dysfunction**	28 (24,43)	27 (24,32)	26 (23,31.75)	29 (25,37)	30 (25,38)	4.102	0.007	27 (24,32)	29 (25,38)	35 (25,39.75)	6.456	0.002	26.5 (23.75,32)	27 (24,32)	28.5 (24,36.5)	30 (24.25,38)	3.888	0.009
**GDS**	3 (1,5)	2 (1,5)	3 (1,5)	3 (1.25,5.75)	3 (2,6)	2.725	0.044	2 (1,5)	4 (2,6)	5 (3,8)	14.922	<0.001	2 (1,4.75)	3 (1,5)	4 (1,6)	3 (2,6)	3.393	0.018
**NPI**	0 (0,2)	0 (0,1)	0 (0,2)	0 (0,1.77)	0 (0,2)	1.019	0.384	0 (0,1)	0 (0,3)	2 (0,3)	11.405	<0.001	0 (0,1)	0 (0,1)	0 (0,2)	0 (0,2.75)	3.603	0.014

*ARHL, age-related hearing loss; BMI, body mass index; CVD, cardiovascular disease; MMSE, the Mini-Mental Status Exam; MCI, mild cognitive impairment; GDS, the Geriatric Depression Scale; NPI, self-report Neuropsychiatric Inventory Questionnaire. HL 0 = Normal hearing; HL 1 = subclinical hearing loss; HL 2 = mild HL; and HL 3 = moderate or severe HL. Tinnitus 0 = without tinnitus; tinnitus 1 = mild tinnitus; and tinnitus 3 = moderate or disaster tinnitus. The presence of ARHL and/or tinnitus 0 = without ARHL and tinnitus; the presence of ARHL and/or tinnitus 1 = with ARHL; the presence of ARHL and/or tinnitus 2 = with tinnitus; and the presence of ARHL and/or tinnitus 3 = with ARHL and tinnitus. Physical frailty 0 = robust; physical frailty 1 = pre-physical frailty; and physical frailty 2 = physical frailty*.

### Association of ARHL Severity and Physical Frailty With HRQoL

The results of the multiple logistic regression models on the relationships between the severity of ARHL and high HRQoL (low scores) or low subscores in the physical dimensions of HRQoL are shown in Table 2. No significant association between ARHL severity and HRQoL was observed. Physical frailty was significantly associated with low HRQoL. In Model 1, female sex was associated with a significantly lower probability of high HRQoL (odds ratio [OR] = 0.585, 95% confidence interval [CI] = 0.354–0.966) (Table 2). In Model 2, no physical frailty (OR = 11.839, 95% CI = 3.554–39.436], pre-physical frailty (OR = 6.028, 95% CI = 1.724–21.08), and no CVD (OR = 2.007, 95% CI = 1.148–3.51) were associated with significantly higher probability of high HRQOL. In Model 3, lack of physical frailty (OR = 17.385, 95% CI = 2.272–133.058), pre-physical frailty (OR = 9.529, 95% CI = 1.191–76.241), and no CVD (OR = 2.24, 95% CI = 1.142–4.393) were associated with significantly higher probability of high HRQOL. Meanwhile, social dysfunction (OR = 0.893, 95% CI = 0.829–0.962) and depressive symptoms (OR =0.797, 95% CI =0.667–0.952) were associated with significantly lower probability of high HRQoL.

**Table 2:** Multiple logistic regression analysis of the association of ARHL severity with high quality of life (low score) and low subscores in domain-specific quality of life.

	**Low quality of life score**			**Independent living**			**Senses**			**Pain**		
	**Model 1**	**Model 2**	**Model 3**	**Model 1**	**Model 2**	**Model 3**	**Model 1**	**Model 2**	**Model 3**	**Model 1**	**Model 2 OR**	**Model 3**
	**OR (95% CI)**	**OR (95% CI)**	**OR (95% CI)**	**OR (95% CI)**	**OR (95% CI)**	**OR (95% CI)**	**OR (95% CI)**	**OR (95% CI)**	**OR (95% CI)**	**OR (95% CI)**	**OR (95% CI)**	**OR (95% CI)**
Stratification of ARHL severity (Ref: 3)												
0							8.423 (4.580, 15.489)***	9.438 (4.892, 18.210)***	10.319 (4.944, 21.536)***			
1							5.423 (2.881, 10.209)***	5.504 (2.733, 11.085)***	5.303 (2.423, 11.610)***			
2							1.308 (0.726, 2.355)	1.494 (0.794, 2.811)	**1.818** **(0.898, 3.680)**			
Age				0.952 (0.920, 0.985)**								
Female (ref: male)	0.585 (0.354, 0.966)*			0.602 (0.375, 0.966)*								
Education level											0.926 (0.869, 0.986)*	0.888 (0.824, 0.957)**
Social dysfunction			0.893 (0.829, 0.962)**			0.950 (0.910, 0.991)*			0.922 (0.890, 0.955)***			0.949 (0.914, 0.986)**
GDS15			0.797 (0.667, 0.952)*									0.873 (0.778, 0.979)*
NPI												**0.835** **(0.691, 1.008)**
Comorbidity (refer 3)												
0					4.497 (1.642, 12.314)**						2.579 (1.160, 5.732)*	
1					3.755 (1.549, 9.102)**						2.336 (1.221, 4.469)**	
2					2.887 (1.170, 7.122)*						2.029 (1.063, 3.873)*	
CVD (ref: 1)		2.007 (1.148, 3.510)*	2.240 (1.142, 4.393)*									
Physical frailty (ref:2)												
0		11.839 (3.554, 39.436)***	17.385 (2.272, 133.058)**		3.739 (1.763, 7.928)***	5.605 (2.237, 14.043)***		2.072 (1.165, 3.685)*			4.184 (2.217, 7.897)***	2.904 (1.400, 6.021)**
1		6.028 (1.724, 21.080)**	9.529 (1.191, 76.241)*		1.497 (0.653, 3.431)	1.623 (0.582, 4.529)		2.028 (1.099, 3.742)*			3.191 (1.652, 6.165)***	2.221 (1.030, 4.790)*

OR = odds ratios; CI = confidence intervals; ARHL, age-related hearing loss; CVD, cardiovascular disease; GDS, the Geriatric Depression Scale; NPI, self-report Neuropsychiatric Inventory Questionnaire. Model 1 is adjusted for age, gender, education, and social dysfunction. Model 2 is adjusted for covariates in model 1 and health-related factors, including self-reported smoking, alcohol intake, BMI, chronic comorbidities, chronic diseases, physical frailty, cognition based on normative z-scores of neuropsychological test battery. Model 3 is adjusted for covariates in model 2 and GDS, NPI and social dysfunction. HL 0 = Normal hearing; HL 1 = subclinical hearing loss; HL 2 = mild HL; and HL 3 = moderate or severe HL. CVD 0 = without CVD; CVD 1 = with CVD. Physical frailty 0 = robust; physical frailty 1 = pre-physical frailty; and physical frailty 2 = physical frailty. *p < 0.05; **p < 0.01; and ***p < 0.001; bold values denote marginally statistical significance.

For the physical dimensions, after adjusting for all the covariates in Model 3, absence of ARHL (OR = 10.319, 95% CI = 4.944–21.536) and subclinical ARHL (OR = 5.303, 95% CI = 2.432–11.61) were significantly associated with a higher probability of better HRQoL in senses domain. Meanwhile, mild ARHL (OR = 1.818, 95% CI = 0.898–3.68) showed a marginal association. In Model 3, physical frailty had no effect on senses, while social dysfunction (OR = 0.922, 95% CI = 0.89–0.955) was associated with significantly worse senses. Lack of physical frailty (OR = 5.605, 2.904; 95% CI = 2.237–14.043, 1.400–6.021, respectively) and pre-physical frailty (OR = 1.623, 2.221; 95% CI = 0.582–4.529, 1.03–4.79, respectively) were associated with significantly higher probability of independence and less pain after adjusting for all the covariates (Table 2). Other independent determinants were social dysfunction for independent living and psychosocial variables and education levels for pain.

Age-related hearing loss severity showed no significant association with all the five psychosocial dimensions (Table 3). No physical frailty was associated with significantly higher happiness (OR = 2.510, 95% CI = 1.109–5.682) and coping (OR = 3.637; 95% CI: 1.032–12.821) in Model 3, and better mental health (OR = 2.581, 95% CI = 1.179–5.647) in Model 2, but not associated with self-worth and relationship. No comorbidity was associated with significantly higher probability of better mental health (OR = 5.082, 95% CI =1.84–14.042), coping (OR = 7.51, 95% CI = 1.937–29.112) in Model 2, and relationships in Models 2 (OR = 6.162, 95% CI = 2.414–15.732) and 3 (OR = 4.046, 95% CI = 1.323–12.374). Depressive symptoms were associated with a significantly lower probability of better functioning across psychosocial dimensions, while social dysfunction was associated with lower mental health, happiness, and relationships.

**Table 3:** Multiple logistic regression analysis of the association of ARHL severity and psychological dimension of HRQoL.

	**Mental health**			**Happiness**			**Self-worth**			**Coping**			**Relationships**		
	**Model 1**	**Model 2**	**Model 3**	**Model 1**	**Model 2**	**Model 3**	**Model 1**	**Model 2**	**Model 3**	**Model 1**	**Model 2**	**Model 3**	**Model 1**	**Model 2**	**Model 3**
	**OR (95% CI)**	**OR (95% CI)**	**OR (95% CI)**	**OR (95% CI)**	**OR (95% CI)**	**OR (95% CI)**	**OR (95% CI)**	**OR (95% CI)**	**OR (95% CI)**	**OR (95% CI)**	**OR (95% CI)**	**OR (95% CI)**	**OR (95% CI)**	**OR (95% CI)**	**OR (95% CI)**
Age		1.080 (1.035,1.126)***	1.097 (1.041, 1.156)**							0.945 (0.902, 0.990)*					
Education level		0.922 (0.857, 0.992)*	0.893 (0.812, 0.982)*												
Smoking ( refer 2)															
0			0.181 (0.061, 0.539)**												
1			0.136 (0.024, 0.773)*												
Social dysfunction			0.836 (0.767, 0.910)***			0.937 (0.893, 0.984)**									0.877 (0.817, 0.941)***
GDS15			0.724 (0.594, 0.882)**			0.835 (0.731, 0.955)**			0.784 (0.673, 0.913)**			0.743 (0.603, 0.915)**			0.839 (0.717, 0.981)*
Comorbidity (refer 3)															
0		5.082 (1.840, 14.042)**									7.510 (1.937, 29.112)**			6.162 (2.414, 15.732)***	4.046 (1.323, 12.374)*
1		3.502 (1.466, 8.365)**									**3.231** **(0.885, 11.795)**			2.610 (1.115, 6.108)*	1.375 (0.486, 3.889)
2		1.867 (0.760, 4.586)									1.634 (0.406, 6.585)			**2.175** **(0.912, 5.185)**	1.725 (0.617, 4.828)
CVD (ref: 1)			2.139 (1.064, 4.299)*												
Physical frailty (ref: 2)															
0		2.581 (1.179, 5.647)*			3.164 (1.675, 5.977)***	2.510 (1.109, 5.682)*					3.206 (1.045, 9.835)*	3.637 (1.032, 12.821)*			
1		1.466 (0.649, 3.312)			1.393 (0.684, 2.835)	1.341 (0.552, 3.258)					1.435 (0.410, 5.023)	1.085 (0.247, 4.765)			

OR, odds ratios; CI, confidence intervals; ARHL, age-related hearing loss; CVD, cardiovascular disease; GDS, the Geriatric Depression Scale. Model 1 is adjusted for mobility CF, non-mobility CF, cognitive decline, and age. Model 1 is adjusted for age, gender, education, and social dysfunction. Model 2 is adjusted for covariates in model 1 and health-related factors, including self-reported smoking, alcohol intake, BMI, chronic comorbidities, chronic diseases, physical frailty, cognition based on normative z-scores of neuropsychological test battery. Model 3 is adjusted for covariates in model 2 and GDS, NPI and social dysfunction. Smoking 0 = never smoking; smoking 1 = ever smoking; smoking 2 = current smoking. Comorbidity 0 = without chronic disease; comorbidity 1 = with 1 chronic disease; comorbidity 2 = with 2 chronic diseases; comorbidity 3 = with more than 2 chronic diseases. CVD 0 = without CVD; CVD 1 = with CVD. Physical frailty 0 = robust; physical frailty 1 = pre-physical frailty; and physical frailty 2 = physical frailty. *p < 0.05; **p < 0.01; and ***p < 0.001; bold values denote marginally statistical significance.

### Association of Physical Frailty and Tinnitus Severity With HRQoL

The results of the multiple regression models on the relationships between tinnitus severity and HRQoL or physical dimensions of HRQoL are shown in Table 4. Absence of tinnitus (OR = 8.014, 95% CI = 1.04–61.722) and mild tinnitus (OR = 10.024, 95% CI = 1.25–80.384) were associated with significantly higher probability of high HRQoL in Model 2, but not in Model 3. Lack of physical frailty (OR = 16.437, 95% CI = 2.146–125.896) and pre-physical frailty (OR = 8.945, 95% CI = 1.118–71.593) were associated with significantly higher probability of high HRQoL in Model 3. Social dysfunction and depressive symptoms were also independent risk factors of high HRQoL. Absence of tinnitus and mild tinnitus were associated with significantly better independent living (OR = 11.904 and 5.354, 95% CI = 1.529–92.697 and 0.631–45.42, respectively) and senses (OR = 12.502 and 7.293, 95% CI = 2.765–56.528 and 1.529–34.797, respectively) in Model 3. Furthermore, absence of tinnitus was associated with significantly lower odds of pain in Models 1 (OR = 3.507, 95% CI = 1.494–8.230) and 2 (OR = 2.639, 95% CI = 1.082–6.434,), but not in Model 3 after adjusting for additional psychosocial covariates. Absence of physical frailty (OR = 6.999 and 3.343, 95% CI = 2.742–17.865 and 1.593–7.018, respectively) was associated with a significantly higher probability of better independent living and lower probability of pain in Model 3, and pre-physical frailty (OR = 2.539, CI = 1.169–5.513) was associated with significantly lower probability of pain in Model 3 after adjusting for all the covariates. CVD was an independent determinant of independent living; age and social dysfunction were independent determinants of senses; and education level, social dysfunction, and depressive symptoms were independent determinants of pain (Table 4).

**Table 4:** Multiple logistic regression analysis of the association of tinnitus severity with high quality of life (low scores) and low subscores of domain-specific quality of life.

	**Low quality of life**			**Independent living**			**Senses**			**Pain**		
	**Model 1 OR (95% CI)**	**Model 2 OR (95% CI)**	**Model 3 OR (95% CI)**	**Model 1 OR (95% CI)**	**Model 2 OR (95% CI)**	**Model 3 OR (95% CI)**	**Model 1 OR (95% CI)**	**Model 2 OR (95% CI)**	**Model 3 OR (95% CI)**	**Model 1 OR (95% CI)**	**Model 2 OR (95% CI)**	**Model 3 OR (95% CI)**
Stratification of tinnitus (refer 2)												
0	10.754 (1.430, 80.889)*	8.014 (1.040, 61.722)*		17.312 (2.299, 130.367)**	14.287 (1.875, 108.853)**	11.904 (1.529, 92.697)*	10.432 (3.571, 30.480)***	11.992 (3.528, 40.761)***	12.502 (2.765, 56.528)***	3.507 (1.494, 8.230)**	2.639 (1.082, 6.434)*	
1	10.852 (1.394, 84.498)*	10.024 (1.250, 80.384)*		10.454 (1.335, 81.870)*	8.388 (1.048, 67.123)*	5.354 (0.631, 45.420)*	4.887 (1.596, 14.968)**	6.521 (1.830, 23.238)**	7.293 (1.529, 34.797)*	1.967 (0.786, 4.925)	1.489 (0.564, 3.930)	
Age	0.962 (0.926, 1.00)*			0.945 (0.911, 0.980)**			0.951 (0.923, 0.981)***	0.959 (0.929, 0.990)**	0.936 (0.903, 0.971)***			
Female (refer male)	0.523 (0.312, 0.877)*			0.575 (0.353, 0.937)*								
Education level										0.942 (0.890, 0.998)*	0.918 (0.862, 0.978)**	0.901 (0.838, 0.970)**
Social dysfunction			0.892 (0.828, 0.961)**						0.921 (0.890, 0.953)***			0.951 (0.916, 0.988)**
GDS15			0.797 (0.667, 0.953)*									0.849 (0.757, 0.951)**
Cognition (refer 2)												
0								2.468 (1.418, 4.295)***				
1								1.922 (1.030, 3.585)*				
CVD (ref: 1)		2.170 (1.232, 3. 821)**	2.236 (1.140, 4.384)*		1.987 (1.180, 3.345)**	1.964 (1.084, 3.557)*						
Physical frailty (refer 2)												
0		8.617 (2.957, 25.110)***	16.437 (2.146, 125.896)**		4.061 (1.978, 8.335)***	6.999 (2.742, 17.865)***					4.878 (2.612, 9.109)***	3.343 (1.593, 7.018)***
1		4.076 (1.317, 12.613)*	8.945 (1.118, 71.593)*		1.251 (0.553, 2.831)	1.698 (0.597, 4.826)					3.156 (1.631, 6.107)***	2.539 (1.169, 5.513)*

Model 1 is adjusted for age, gender, education, and social dysfunction. Model 2 is adjusted for covariates in model 1 and health-related factors, including self-reported smoking, alcohol intake, BMI, chronic comorbidities, chronic diseases, physical frailty, cognition based on normative z-scores of neuropsychological test battery. Model 3 is adjusted for covariates in model 2 and GDS, NPI and social dysfunction. Tinnitus 0 = without tinnitus; tinnitus 1 = mild tinnitus; and tinnitus 3 = moderate or disaster tinnitus. CVD 0 = without CVD; CVD 1 = with CVD. Physical frailty 0 = robust; physical frailty 1 = pre-physical frailty; and physical frailty 2 = physical frailty. *p < 0.05; **p < 0.01; and ***p < 0.001.

Among the five psychosocial dimensions, depressive symptoms were independent risk factors for worse psychosocial dimensions, and social dysfunction was an independent risk factor for worse mental health, happiness, and relationship. Tinnitus severity was not directly associated with the psychosocial dimensions (Table 5). Absence of physical frailty was associated with significantly higher probability of better mental health (OR = 2.664, 95% CI = 1.212–5.856) and happiness (OR = 2.906, 95% CI = 1.558–5.42) in Model 2, and with happiness (OR = 2.427, 95% CI = 1.069–5.509) and coping (OR = 3.584, 95% CI = 1.016–12.638) in Model 3 (Table 5). A lack of or presence of one comorbidity was associated with a significantly higher probability of high HRQoL in the psychosocial dimensions, including mental health, coping, and relationships in Model 2, and no comorbidity was still associated with a significantly higher probability of high HRQoL in the domain of relationships.

**Table 5:** Multiple logistic regression analysis of the association of tinnitus severity and psychological dimension of HRQoL.

	**Mental health**			**Happiness**			**Self-worth**			**Coping**			**Relationships**		
	**Model 1 OR (95% CI)**	**Model 2 OR (95% CI)**	**Model 3 OR (95% CI)**	**Model 1 OR (95% CI)**	**Model 2 OR (95% CI)**	**Model 3 OR (95% CI)**	**Model 1 OR (95% CI)**	**Model 2 OR (95% CI)**	**Model 3 OR (95% CI)**	**Model 1 OR (95% CI)**	**Model 2 OR (95% CI)**	**Model 3 OR (95% CI)**	**Model 1 OR (95% CI)**	**Model 2 OR (95% CI)**	**Model 3 OR (95% CI)**
Age		1.083 (1.038, 1.129)***	1.095 (1.040, 1.154)***							0.953 (0.910, 0.997)*					
Education level		0.918 (0.853, 0.987)*	0.896 (0.814, 0.985)*												
Smoking (refer 2)															
0			0.179 (0.060, 0.533)**												
1			0.138 (0.024, 0.780)*												
Social dysfunction			0.839 (0.770, 0.914)***			0.936 (0.891, 0.982)**									0.880 (0.820, 0.944)***
GDS15			0.721 (0.591, 0.879)***			0.836 (0.731, 0.956)**			0.784 (0.673, 0.913)**			0.743 (0.603, 0.915)**			0.833 (0.711, 0.976)*
Comorbility (refer 3)															
0		5.263 (1.903, 14.554)***									10.629 (2.829, 39.927)***			5.871 (2.275, 15.150)***	4.058 (1.328, 12.403)*
1		3.488 (1.456, 8.359)**									4.636 (1.310, 16.408)*			2.553 (1.076, 6.053)*	1.295 (0.454, 3.690)
2		2.016 (0.826, 4.923)									2.195 (0.566, 8.508)			**2.107 (0.878, 5.058)**	1.721 (0.615, 4.811)
CVD (ref: 1)			2.062 (1.022, 4.161)*												
Physical frailty (refer 2)															
0		2.664 (1.212, 5.856)*			2.906 (1.558, 5.420)***	2.427 (1.069, 5.509)*						3.584 (1.016, 12.638)*			
1		1.514 (0.667, 3.438)			1.222 (0.604, 2.474)	1.287 (0.529, 3.133)						1.059 (0.241, 4.651)			

OR, odds ratios; CI, confidence intervals; CVD, cardiovascular disease; GDS, the Geriatric Depression Scale. Model 1 is adjusted for age, gender, education, and social dysfunction. Model 2 is adjusted for covariates in model 1 and health-related factors, including self-reported smoking, alcohol intake, BMI, chronic comorbidities, chronic diseases, physical frailty, cognition based on normative z-scores of neuropsychological test battery. Model 3 is adjusted for covariates in model 2 and GDS, NPI and social dysfunction. Comorbidity 0 = without chronic disease; comorbidity 1 = with 1 chronic disease; comorbidity 2 = with 2 chronic diseases; comorbidity 3 = with more than 2 chronic diseases. Smoking 0 = never smoking; smoking 1 = ever smoking; smoking 2 = current smoking. CVD 0 = without CVD; CVD 1 = with CVD. Physical frailty 0 = robust; physical frailty 1 = pre-physical frailty; and physical frailty 2 = physical frailty. ^*^p < 0.05; ^**^p < 0.01; and ^***^p < 0.001; bold values denote marginally statistical significance.

### Association of Physical Frailty and the Presence of ARHL and/or Tinnitus With HRQoL

The relationships of presence of ARHL and/or tinnitus with HRQoL or physical dimensions of HRQoL in the different multiple regression models are shown in Table 6. The presence of ARHL and/or tinnitus was not significantly associated with HRQoL or pain as a physical dimension. The lack of ARHL and tinnitus and the presence of ARHL and/or tinnitus were associated with significantly low HRQoL subscores of physical dimensions of independent living (OR = 2.372, 95% CI = 1.01–5.573; OR = 2.786, 95% CI = 1.337–5.804; and OR = 3.551, 95% CI = 1.132–11.14, respectively) and sense (OR = 11.843, 95% CI = 5.477–25.605; OR = 2.56, 95% CI = 1.404–4.667; OR = 4.712, 95% CI = 1.753–12.665, respectively) after adjusting for all the covariates. No physical frailty and pre-physical frailty were associated with significantly higher probability of low HRQoL scores (OR = 17.385, 95% CI = 2.272–133.058 and OR = 9.529, 95% CI = 1.191–76.241, respectively) and low pain (OR = 2.901, 95% CI = 1.4–6.021 and OR = 2.221, 95% CI = 1.03–4.79, respectively) in Model 3. Patients without physical frailty were significantly more likely to have better independent living (low subscores) (OR = 5.864, 95% CI = 2.297–14.97) in Model 3.

**Table 6:** Multiple logistic regression analysis of the association of the presence of ARHL and/or tinnitus with high quality of life (low scores) and low subscores of domain-specific quality of life.

	**Low quality of life**			**Independent living**			**Senses**			**Pain**		
	**Model 1 OR (95% CI)**	**Model 2 OR (95% CI)**	**Model 3 OR (95% CI)**	**Model 1 OR (95% CI)**	**Model 2 OR (95% CI)**	**Model 3 OR (95% CI)**	**Model 1 OR (95% CI)**	**Model 2 OR (95% CI)**	**Model 3 OR (95% CI)**	**Model 1 OR (95% CI)**	**Model 2 OR (95% CI)**	**Model 3 OR (95% CI)**
Stratification of ARHL and/or tinnitus (Ref: 3)												
0				1.945 (1.001, 3.780)*	2.249 (1.081, 4.679)*	2.372(1.010, 5.573)*	9.774 (5.174, 18.463)***	9.600 (4.908, 18.775)***	11.843 (5.477, 25.605)***			
1				2.146 (1.221, 3.773)**	2.495 (1.324, 4.701)**	2.786(1.337, 5.804)**	2.550 (1.568, 4.145)***	2.702 (1.604, 4.552)***	2.560 (1.404, 4.667)**			
2				1.963 (0.798, 4.827)	**2.607 (0.996, 6.824)**	3.551 (1.132, 11.140)*	3.457 (1.541, 7.803)**	3.598 (1.541, 8.405)**	4.712 (1.753, 12.665)**			
Age	0.962 (0.926, 0.998)*			0.948 (0.915, 0.983)**			0.958 (0.928, 0.988)**	0.965 (0.934, 0.997)*	0.941 (0.906, 0.977)**			
Female (Ref: male)	0.548 (0.328, 0.917)*			0.599 (0.370, 0.969)*								
Education level											0.923 (0.866, 0.984)*	0.888 (0.824, 0.957)**
Social dysfunction			0.893 (0.829, 0.962)**			**0.958 (0.918, 1.001)**			0.919 (0.886, 0.953)***			0.949 (0.914, 0.986)**
GDS15			0.797 (0.667, 0.952)*									0.873 (0.778, 0.979)*
NPI												**0.835 (0.691, 1.008)**
Cognition (Ref 2)												
0								2.100 (1.170, 3.771)*				
1								1.942 (1.012, 3.727)*				
Comorbidity (Ref 3)												
0											2.578 (1.159, 5.734)*	
1											2.332 (1.219, 4.463)*	
2											1.977 (1.034, 3.781)*	
CVD (Ref: 1) 0		2.080 (1.184, 3.652)*	2.240 (1.142, 4.393)*		1.928 (1.144, 3.250)*	1.961 (1.080, 3.562)*						
Physical frailty (Ref: 2)												
0		11.835 (3.552, 39.437)***	17.385 (2.272, 133.058)**		4.648 (2.215, 9.755)***	5.864 (2.297, 14.970)***					4.189 (2.218, 7.909)***	2.901 (1.400, 6.021)**
1		5.684 (1.617, 19.976)**	9.529 (1.191, 76.241)*		1.464 (0.631, 3.395)	1.620 (0.565, 4.644)					3.129 (1.617, 6.053)***	2.221 (1.030, 4.790)*

OR, odds ratios; CI, confidence intervals; GDS, the Geriatric Depression Scale; NPI, self-report Neuropsychiatric Inventory Questionnaire. Model 1 is adjusted for age, gender, education, and social dysfunction. Model 2 is adjusted for covariates in model 1 and health-related factors, including self-reported smoking, alcohol intake, BMI, chronic comorbidities, chronic diseases, physical frailty, cognition based on normative z-scores of neuropsychological test battery. Model 3 is adjusted for covariates in model 2 and GDS, NPI and social dysfunction. The presence of ARHL and/or tinnitus 0 = without ARHL and tinnitus; the presence of ARHL and/or tinnitus 1 = with ARHL; the presence of ARHL and/or tinnitus 2 = with tinnitus; and the presence of ARHL and/or tinnitus 3 = with ARHL and tinnitus. Cognition 0 = normal cognition; cognition 1 = pre-MCI; cognition 2 = MCI. CVD 0 = without CVD; CVD 1 = with CVD. Physical frailty 0 = robust; physical frailty 1 = pre-physical frailty; and physical frailty 2 = physical frailty. *p < 0.05; **p < 0.01; and ***p < 0.001; bold values denote marginally statistical significance.

The presence of ARHL and/or tinnitus was not associated with any of the five psychosocial HRQoL domains (Table 7). Those without physical frailty showed a significantly higher probability of high HRQoL (low subscores) in mental health (OR = 2.595, 95% CI = 1.185–5.681) after adjusting for the covariates in Model 2 and in happiness (OR = 2.51, 95% CI = 1.109–5.682) and coping (OR = 3.637, 95% CI = 1.032–12.821) after adjusting for all the covariates in Model 3. No comorbidity or only one comorbidity was associated with a significantly higher probability of high HRQoL (low subscores) in mental health (OR = 5.1, 95% CI = 1.845–14.092; OR = 3.513, 95% CI = 1.471–8.392, respectively), coping (OR = 7.512, 95% CI = 1.938–29.114; OR = 3.233, 95% CI = 0.886–11.8, *p* = 0.076, respectively) and relationships (OR = 6.162, 95% CI = 2.414–15.732; OR = 2.61, 95% CI = 1.115–6.108, respectively) after adjusting for the covariates in Model 2 and in relationships (OR = 4.046, 95% CI = 1.323–12.374) after adjusting for all the covariates in Model 3. Depressive symptoms were the major determinant of all the five psychosocial dimensions, and social dysfunction was an important determinant of mental health, happiness, and relationships. Age, education level, smoking, and CVD were also independent risk factors for mental health after adjusting for all the covariates.

**Table 7:** Multiple logistic regression analysis of the association of the presence of ARHL and/or tinnitus and psychological dimension of HRQoL.

	**Mental health**			**Happiness**			**Self-worth**			**Coping**			**Relationship**		
	**Model 1 OR (95% CI)**	**Model 2 OR (95% CI)**	**Model 3 OR (95% CI)**	**Model 1 OR (95% CI)**	**Model 2 OR (95% CI)**	**Model 3 OR (95% CI)**	**Model 1 OR (95% CI)**	**Model 2 OR (95% CI)**	**Model 3 OR (95% CI)**	**Model 1 OR (95% CI)**	**Model 2 OR (95% CI)**	**Model 3 OR (95% CI)**	**Model 1 OR (95% CI)**	**Model 2 OR (95% CI)**	**Model 3 OR (95% CI)**
Age		1.081 (1.036, 1.128)***	1.097 (1.041, 1.156)***							0.945 (0.902, 0.991)*					
Education level		0.923 (0.858, 0.993)*	0.893 (0.812, 0.982)*												
Smoking (Ref 2)															
0			0.181 (0.061, 0.539)**												
1			0.136 (0.024, 0.773)*												
Social dysfunction			0.836 (0.767, 0.910)***			0.937 (0.893, 0.984)**									0.877 (0.817, 0.941)***
GDS15			0.724 (0.594,0.882)***			0.835 (0.731, 0.955)**			0.784 (0.673, 0.913)**			0.743 (0.603, 0.915)**			0.839 (0.717, 0.981)*
Comorbidity (Ref 3)															
0		5.100 (1.845, 14.092)**									7.512 (1.938, 29.114)**			6.162 (2.414, 15.732)***	4.046 (1.323, 12.374)*
1		3.513 (1.471, 8.392)**									**3.233 (0.886, 11.800)**			2.610 (1.115, 6.108)*	1.375(0.486, 3.889)
2		1.897 (0.772, 4.660)									1.646 (0.408, 6.631)			2.066 (0.862, 4.955)	1.725 (0.617, 4.828)
CVD (Ref: 1)			2.139(1.064, 4.299)*												
physical frailty (Ref: 2)															
0		2.595 (1.185, 5.681)*			3.164 (1.675, 5.977)***	2.510 (1.109, 5.682)*					3.208 (1.046, 9. 840)*	3.637 (1.032, 12.821)*			
1		1.489 (0.658, 3.368)			1.332 (0.651, 2.726)	1.341 (0.552, 3.258)					1.444 (0.413, 5.055)	1.085 (0.247, 4.765)			

OR, odds ratios; CI, confidence intervals; GDS, the Geriatric Depression Scale; NPI, self-report Neuropsychiatric Inventory Questionnaire. Model 1 is adjusted for age, gender, education, and social dysfunction. Model 2 is adjusted for covariates in model 1 and health-related factors, including self-reported smoking, alcohol intake, BMI, chronic comorbidities, chronic diseases, physical frailty, cognition based on normative z-scores of neuropsychological test battery. Model 3 is adjusted for covariates in model 2 and GDS, NPI and social dysfunction. Smoking 0 = never smoking; smoking 1 = ever smoking; smoking 2 = current smoking. CVD 0 = without CVD; CVD 1 = with CVD. Physical frailty 0 = robust; physical frailty 1 = pre-physical frailty; and physical frailty 2 = physical frailty. *p < 0.05; **p < 0.01; and ***p < 0.001; bold values denote marginally statistical significance.

## Discussion

The impact of the presence and severity of ARHL and tinnitus, ARHL, tinnitus, and physical frailty on the HRQoL and its specific domains in community-dwelling older adults is yet to be clarified to date. This study found an association of ARHL severity with the senses domain of HRQoL, and the association remains significant after adjusting for demographic, health-related, and psychosocial covariates. Tinnitus severity is negatively associated with HRQoL and with three physical dimensions, but the association is no longer significant for HRQoL and pain after adjusting for all the covariates in Model 3. The presence of ARHL and/or tinnitus is only significantly associated with independent living and senses, and the association remains significant after adjusting for all the covariates. The severity of physical frailty is significantly associated with worse HRQoL (high scores) and independent living and pain (high subscores), and the associations remain significant after adjusting for all the confounders. Moreover, the severity of physical frailty is also significantly associated with the psychosocial domains of mental health, happiness, and coping, and the association is only decreased for mental health after adjusting for the psychosocial covariates in Model 3. The use of a multidimensional HRQoL questionnaire and the three models, with successive adjustments for different covariates, provided original information on these relationships.

These results extend our understanding of the association among the severity of ARHL (8, 9), tinnitus (10, 18), physical frailty (13, 14, 26), and the presence of ARHL and/or tinnitus (17) with HRQoL. Furthermore, these findings provide more evidence on the interaction among ARHL, tinnitus, and physical frailty in their relationship with HRQoL after adjusting for demographic, health-related, and psychosocial factors. Physical frailty coexisting with ARHL, tinnitus, and ARHL with tinnitus had a stronger effect on the overall HRQoL. Several previous studies have indicated that ARHL (8, 9, 28, 29) and/or tinnitus (10, 30) were negatively associated with HRQoL. However, we found no significant association among ARHL severity, the presence of ARHL and/or tinnitus, and HRQoL, and the association between tinnitus severity and HRQoL was also no longer significant after adjustment for psychosocial factors. The difference in our results, in comparison with previous findings, may be due to the different assessment tools used for HRQoL and various covariates. Physical frailty has been shown to be an important determinant of HRQoL in numerous studies (13, 14), and consistent findings were obtained in this study. Physical frailty remained significantly associated with HRQoL after adjusting for all the covariates, such as the severity of ARHL and tinnitus and the presence of ARHL and/or tinnitus. Moreover, those with physical frailty had a significantly higher probability of poor HRQoL than those with pre-physical frailty.

The effects of age-related auditory disorders (ARHL and tinnitus) on the specific domains of HRQoL were significantly different from those of physical frailty. Previous studies have indicated that ARHL, tinnitus, and the presence of ARHL and/or tinnitus were significantly associated with physical function (7), frailty phenotypes (3), cognition (31, 32), social dysfunction (33), and psychological disorders (e.g., depressive symptoms and anxiety) (10, 11, 24, 34). However, this study found no significant associations between these age-related auditory disorders and the psychosocial dimensions of HRQoL. Among the three physical dimensions, ARHL severity was only significantly associated with senses. This is consistent with a previous finding that hearing loss is inversely associated with physical health scores after adjusting for sociodemographic and health-related factors (35). Furthermore, we found that tinnitus is significantly associated with independent living, senses, and pain after adjusting for the demographic and health-related factors. However, the association between tinnitus severity and pain was no longer significant after adjusting for the psychosocial factors. A large-scale population-based study reported that people with increasing tinnitus severity had worse physical, pain, vitality, and mental health HRQoL (18). In another study, depression-and anxiety-mediated chronic tinnitus caused impairments in mental and physical HRQoL in women and men, respectively (10).

Our study also showed that psychosocial factors, such as social dysfunction and depressive symptoms, were independent determinants of the psychosocial dimensions of HRQoL. In a large cross-sectional population-based study of individuals aged >19 years, hearing loss with tinnitus had a significant impact on all five dimensions (mobility, self-care, usual activities, pain/discomfort, and anxiety/depression) of HRQoL (17). In contrast, our results showed that the presence of ARHL and/or tinnitus was significantly associated with independent living and senses in physical dimensions, but not with psychosocial dimensions after adjusting for all the covariates.

Several studies have shown that frailty, including physical frailty, is significantly associated with worse HRQoL in the community-dwelling older people (13, 14). Among Fried's frailty criteria, “slowness” had the strongest correlation with worse HRQoL after adjusting for the age, sex, and socioeconomic and health covariates (26). Our results revealed that physical frailty has different effects on the physical and psychosocial dimensions of HRQoL. It was not only significantly associated with independent living and pain in the physical dimensions, but also with happiness and coping in the psychosocial dimensions. Participants with physical frailty had worse HRQoL in these dimensions than those with pre-physical frailty. Given that gait and ARHL are important contributors to frailty and the importance of sensory and locomotion domains in intrinsic capacity (6, 36), the different effects of multiple sensory and functional mobility impairments on HRQoL and specific domains need to be further investigated in the future.

Aside from ARHL, tinnitus, physical frailty, education level, BMI, alcohol intake, disability in basic activities of daily living, multicomorbidity, chronic diseases, cognition, and depressive symptoms also influence HRQoL (9, 10, 17, 26, 35). In this study, age and education level were independent determinants of the senses and pain domains of the physical dimension, respectively. Social dysfunction was an independent risk factor for overall HRQoL; senses and pain in the physical dimension; and mental health, happiness, and relationships in the psychosocial dimension. Depressive symptoms were independent determinants of overall HRQoL, physical pain, and all the five psychosocial dimensions. CVD was a risk factor for worse HRQoL, independent living in the physical dimension, and mental health in the psychosocial dimension. In addition, the number of comorbidities was associated with mental health, coping, and relationships, especially relationships in the psychosocial dimension.

To our best knowledge, this cross-sectional study is the first to report the different effects of the severity of ARHL, tinnitus, and physical frailty, and the presence of ARHL and/or tinnitus on overall HRQoL and its specific domains. The use of a multidimensional HRQoL instrument, in combination with several self-report and objective covariates, in the assessment improved the robustness of the data collected. However, there are also some limitations to this study. First, participation was voluntary, which could have led to selection bias. Second, the cross-sectional design limited the analysis of causal relationships. For example, ARHL and tinnitus are associated with depressive symptoms (3, 9, 10, 16–18, 28, 29, 34) and social dysfunction (3, 16, 33), which are independent determinants of HRQoL and specific domains. However, we failed to identify a significant association between ARHL/tinnitus and HRQoL/psychosocial dimensions of HRQoL. Moreover, it is difficult to determine whether ARHL, tinnitus, and physical frailty are causes or consequences of poor HRQoL and its specific domains. Finally, senses in the physical HRQoL dimension included few parameters of auditory-related QoL, such as sound localization, hearing in noise, sound clarity, and pitches (16). However, these are crucial for assessing HRQoL after cochlear implant or hearing aid use. Longitudinal studies are needed to understand the causal relationships among ARHL, tinnitus, physical frailty, and HRQoL.

In conclusion, ARHL severity is an independent determinant of senses, while tinnitus severity is an independent determinant of independent living and senses in physical HRQoL. Physical frailty is an independent determinant of overall HRQoL, of independent living and pain in the physical dimension, and of happiness and coping in the psychosocial dimension.

## Data Availability Statement

The original contributions presented in the study are included in the article/supplementary material, further inquiries can be directed to the corresponding authors.

## Ethics Statement

The studies involving human participants were reviewed and approved by the Ethics Committee of Huadong Hospital, Fudan University. The patients/participants provided their written informed consent to participate in this study.

## Author Contributions

QR, ZY, and ZH conceived and designed the study. QR wrote the article. WZ, JR, RZ, MZ, XH, and ZY conducted the investigation and data analysis. All authors contributed to the article and approved the submitted version.

## Funding

This study was supported by the Medical Science and Technology Support Project of Shanghai Science and Technology Commission (Grant Nos: 18411962200 and 20Y11902300), Shanghai Municipal Key Clinical Specialty (Grant No: shslczdzk02801), and the National Key Research and Innovation Project (2018YFC2002000).

## Conflict of Interest

The authors declare that the research was conducted in the absence of any commercial or financial relationships that could be construed as a potential conflict of interest.

## Publisher's Note

All claims expressed in this article are solely those of the authors and do not necessarily represent those of their affiliated organizations, or those of the publisher, the editors and the reviewers. Any product that may be evaluated in this article, or claim that may be made by its manufacturer, is not guaranteed or endorsed by the publisher.

## References

[1] World Health Organization. Deafness and hearing Loss. (2019). Available online at: https://www.who.int/news-room/fact-sheets/detail/deafness-and-hearingloss (accessed 3 January 2020).

[2] Hoogendijk EO Afilalo J Ensrud KE Kowal P Onder G Fried LP. Frailty: implications for clinical practice and public health. Lancet. (2019) 394:1365–75. 10.1016/S0140-6736(19)31786-6 31609228

[3] Ruan Q Chen J Zhang R Zhang W Ruan J Zhang M. Heterogeneous influence of frailty phenotypes in age-related hearing loss and tinnitus in Chinese older adults: an explorative study. Front Psychol. (2020) 11:617610. 10.3389/fpsyg.2020.617610 33664689 PMC7921692

[4] Gates GA Mills JH. Presbycusis. Lancet. (2005) 366:1111–20. 10.1016/S0140-6736(05)67423-5 16182900

[5] Mathers C. The global burden of disease: 2004 update. Geneva: WHO (2008). Available online at: http://www.who.int/healthinfo/global_burden_disease/GBD_report_2004update_full.pdf (accessed March 7, 2018)

[6] Panza F Solfrizzi V Logroscino G. Age-related hearing impairment-a risk factor and frailty marker for dementia and AD. Nat Rev Neurol. (2015) 11:166–75. 10.1038/nrneurol.2015.12 25686757

[7] Kamil RJ Betz J Powers BB Pratt S Kritchevsky S Ayonayon HN. Association of HEARing Impairment with Incident Frailty and Falls in Older Adults. J Aging Health. (2016) 28:644–60. 10.1177/0898264315608730 26438083 PMC5644033

[8] Strawbridge WJ Wallhagen MI Shema SJ Kaplan GA. Negative consequences of hearing impairment in old age: a longitudinal analysis. Gerontologis. (2000) 40:320–6. 10.1093/geront/40.3.320 10853526

[9] Baek MK Kim YS Kim EY Kim AJ Choi WJ. Health-related quality of life in Korean adults with hearing impairment: the Korea National health and nutrition examination survey 2010 to 2012. PLoS ONE. (2016) 11:e0163999. 10.1371/journal.pone.0163999 27711203 PMC5053436

[10] Boecking B Biehl R Brueggemann P Mazurek B. Health-related quality of life, depressive symptoms, anxiety, and somatization symptoms in male and female patients with chronic tinnitus. J Clin Med. (2021) 10:2798. 10.3390/jcm10132798 34202097 PMC8267833

[11] Park KH Lee SH Koo JW Park HY Lee KY Choi YS Oh KW Lee A Yang JE Woo SY et al. Prevalence and associated factors of tinnitus: data from the Korean National Health and Nutrition Examination survey 2009–2011. J Epidemiol. (2014)24:417–426. 10.2188/jea.JE20140024 24953134 PMC4150014

[12] Collard RM Boter H Schoevers RA Oude Voshaar RC. Prevalence of frailty in community-dwelling older persons: a systematic review. J Am Geriatr Soc. (2012) 60:1487–92. 10.1111/j.1532-5415.2012.04054.x 22881367

[13] Crocker TF Brown L Clegg A Farley K Franklin M Simpkins S Young J. Quality of life is substantially worse for community-dwelling older people living with frailty: systematic review and meta-analysis. Qual Life Res. (2019) 28:2041–56. 10.1007/s11136-019-02149-1 30875008 PMC6620381

[14] Kojima G Iliffe S Jivraj S Walters K. Association between frailty and quality of life among community-dwelling older people: a systematic review and meta-analysis. J Epidemiol Commun Health. (2016) 70:716–21. 10.1136/jech-2015-206717 26783304

[15] Ciorba A Bianchini C Pelucchi S Pastore A. The impact of hearing loss on the quality of life of elderly adults. Clin Interv Aging. (2012) 7:159–163. 10.2147/CIA.S26059 22791988 PMC3393360

[16] Dixon PR Feeny D Tomlinson G Cushing S Chen JM Krahn MD. Health-related quality of life changes associated with hearing loss. JAMA Otolaryngol Head Neck Surg. (2020) 146:630–8. 10.1001/jamaoto.2020.0674 32407468 PMC7226291

[17] Joo YH Han KD Park KH. Association of HEARing Loss and Tinnitus with Health-Related Quality of Life: the Korea National health and nutrition examination survey. PLoS ONE. (2015) 10:e0131247. 10.1371/journal.pone.0131247 26121026 PMC4488242

[18] Nondahl DM Cruickshanks KJ Dalton DS Klein BE Klein R Schubert CR Tweed TS Wiley TL. The impact of tinnitus on quality of life in older adults. J Am Acad Audiol. (2007)18:257–266. 10.3766/jaaa.18.3.7 17479618

[19] Coary R Skerritt C Carey A Rudd S Shipway D. New horizons in rib fracture management in the older adult. Age Ageing. (2020) 49:161–167. 10.1093/ageing/afz157 31858117

[20] Wu ST Chiu CJ. Age-related trajectories of memory function in middle-aged and olderadults with and without hearing impairment. Neuroepidemiology. (2016) 46:282–9. 10.1159/000445378 27105004

[21] BonfiglioV UmegakiH KuzuyaM. A study on the relationship between cognitive performance, hearing impairment, and frailty in older adults. Dement Geriatr Cogn Disord. (2020) 49:156–62. 10.1159/000507214 32320988

[22] RuanQXiaoFGongKZhangWZhangMRuanJZhangXChenQYuZ. Prevalence of cognitive frailty phenotypes and associated factors in a community-dwelling elderly population. J Nutr Health Aging. (2020) 24:172–80. 10.1007/s12603-019-1286-732003407

[23] Richardson J Iezzi A Khan MA Maxwell A. Validity and reliability of the Assessment of Quality of Life (AQoL)-8D multi-attribute utility instrument. Patient. (2014) 7:85–96. 10.1007/s40271-013-0036-x 24271592 PMC3929769

[24] Golub JS Brewster KK Brickman AM Ciarleglio AJ Kim AH Luchsinger JA Rutherford BR. Subclinical hearing loss is associated with depressive symptoms. Am J Geriatr Psychiatry. (2020) 28:545–556. 10.1016/j.jagp.2019.12.008 31980375 PMC7324246

[25] Ruan Q Xiao F Gong K Zhang W Zhang M Ruan J. Demographically corrected normative Z scores on the neuropsychological test battery in cognitively normal older Chinese adults. Dement Geriatr Cogn Disord. (2020) 49:375–83. 10.1159/000505618 33176318

[26] HenchozYBülaCGuessousISantos-EggimannB. Association between physical frailty and quality of life in a representative sample of community-dwelling Swiss older people. J Nutr Health Aging. (2017) 21:585–92. 10.1007/s12603-016-0772-428448091

[27] Hao QK Li J Dong BR. Chinese experts consensus on assessment and intervention for elderly patients with frailty. Chin J Geriatr. (2017) 36:251–6. 10.3760/cma.j.issn.0254-9026.2017.03.007

[28] Cieśla K Lewandowska M Skarzyński H. Health-related quality of life and mental distress in patients with partial deafness: preliminary findings. Eur Arch Otorhinolaryngol. (2016) 273:767–76. 10.1007/s00405-015-3713-7 26242252 PMC4762916

[29] Ye X Zhu D Chen S He P. The association of hearing impairment and its severity with physical and mental health among Chinese middle-aged and older adults. Health Qual Life Outcomes. (2020) 18:155. 10.1186/s12955-020-01417-w 32456646 PMC7249342

[30] Ukaegbe OC Orji FT Ezeanolue BC Akpeh JO Okorafor IA. Tinnitus and its effect on the quality of life of sufferers: A Nigerian cohort study. Otolaryngol Head Neck Surg. (2017) 157:690–5. 10.1177/0194599817715257 28695761

[31] Loughrey DG Kelly ME Kelley GA Brennan S Lawlor BA. Association of age-related hearing loss with cognitive function, cognitive impairment, and dementia: A systematic review and meta-analysis. JAMA Otolaryngol Head Neck Surg. (2018) 144:115–26. 10.1001/jamaoto.2017.2513 29222544 PMC5824986

[32] Clarke NA Henshaw H Akeroyd MA Adams B Hoare DJ. Associations between subjective tinnitus and cognitive performance: systematic review and meta-analyses. Trends Hear. (2020) 24:2331216520918416. 10.1177/2331216520918416 32436477 PMC7243410

[33] Mick P Kawachi I Lin FR. The association between hearing loss and social isolation in older adults. Otolaryngol Head Neck Surg. (2014) 150:378–84. 10.1177/0194599813518021 24384545

[34] Li CM Zhang X Hoffman HJ Cotch MF Themann CL Wilson MR. Hearing impairment associated with depression in US adults, National Health and Nutrition Examination survey 2005–2010. JAMA Otolaryngol Head Neck Surg. (2014) 140:293–302. 10.1001/jamaoto.2014.42 24604103 PMC4102382

[35] Tsimpida D Kaitelidou D Galanis P. Determinants of health- related quality of life (HRQoL) among deaf and hard of hearing adults in Greece: a cross-sectional study. Arch Public Health. (2018) 76:55. 10.1186/s13690-018-0304-2 30338066 PMC6174559

[36] Cesari M Araujo de Carvalho I Amuthavalli Thiyagarajan J Cooper C Martin FC Reginster JY. Evidence for the domains supporting the construct of intrinsic capacity. J Gerontol A Biol Sci Med Sci. (2018) 73:1653–60. 10.1093/gerona/gly011 29408961

